# ATP-sensitive muscle afferents activate spinal trigeminal neurons with meningeal afferent input in rat – pathophysiological implications for tension-type headache

**DOI:** 10.1186/s10194-016-0668-z

**Published:** 2016-08-26

**Authors:** Moritz Nöbel, Stephan Feistel, Jens Ellrich, Karl Messlinger

**Affiliations:** 1 Institute of Physiology and Pathophysiology, Friedrich-Alexander-University of Erlangen-Nürnberg, Universitätsstr. 17, 91054 Erlangen, Germany; 2 Department of Health Science and Technology, Medical Faculty, Aalborg University, Aalborg, Denmark

**Keywords:** Tension-type headache, Pericranial muscles, Meningeal nociception, α,β-meATP, Local anaesthesia

## Abstract

**Background:**

Tension-type headache and other primary headaches may be triggered or aggravated by disorders of pericranial muscles, which is possibly due to convergent or collateral afferent input from meningeal and muscular receptive areas. In rodent models high extracellular concentrations of ATP caused muscle nociception and central sensitization of second order neurons. In a rat model of meningeal nociception we asked if spinal trigeminal activity induced by ATP can be modulated by local anaesthesia of distinct muscles.

**Methods:**

Ongoing activity was recorded from spinal trigeminal neurons with afferent input from the cranial dura mater, the temporal muscle and neck muscles. The stable ATP analogue α,β-methylene adenosine 5′-triphosphate (α,β-meATP, 10 mM) was injected into the ipsilateral temporal muscle, 30 min later followed by injection of local anaesthetics (lidocaine, 2 %) into the ipsilateral neck muscles and/or the temporal muscle.

**Results:**

Injection of α,β-meATP into the temporal muscle caused progressive increase in ongoing activity of most of the spinal trigeminal neurons within 30 min. Injection of lidocaine into the neck muscles and/or the temporal muscle reduced this activation to previous levels within 10 min.

**Conclusions:**

Distinct spinal trigeminal neurons processing meningeal nociceptive information are under the control of convergent afferent input from several pericranial muscles. Blockade of at least one of these inputs can normalize central trigeminal activity. This may explain why therapeutic manipulations of head muscles can be beneficial in primary headaches.

## Background

Tension-type headache (TTH) and migraine are the most frequent types of primary headache [1, 2]. Psychophysical studies in patients have indicated an important role of neck muscle nociception inducing central sensitization in the pathophysiology of TTH [3, 4], which has been confirmed in animal experiments [5, 6]. Pericranial tenderness and hardness of head and neck muscles are significantly pronounced in TTH and migraine patients compared to healthy volunteers [7–9]. Moreover, pericranial tenderness is positively associated with both the intensity and frequency of TTH [8] and more pronounced in patients with co-occurrence of migraine [10]. Accordingly, pressure pain and tolerance thresholds of head and neck regions are significantly decreased in TTH patients compared with healthy controls [11]. During cervical examination of patients suffering from migraine or TTH patients reported referred pain similar to the headache they usually experienced [12].

Adenosine 5′-triphosphate (ATP) is essential for an effective muscle contraction [13]. Natural concentrations of ATP in muscle cytosol are in the millimolar range buffered by creatine phosphate [14]. Interstitial ATP levels rise measurably dependent on experimental muscle tension [15] and compression [16] but also during natural exercise in humans associated with intensity of muscle contraction [17]. Upon cell damage and inflammation [18] high extracellular levels of ATP may induce purinergic signaling [19] and contribute to muscle pain [6, 20, 21]. In the rat ATP at concentrations present in muscle cells activated the majority of unmyelinated nociceptive and non-nociceptive afferent fibres [22] but high concentrations of ATP may decrease mechanical sensitivity of thin muscle afferents [23].

The stable ATP analogue α,β-methylene adenosine 5′-triphosphate (α,β-meATP) is most frequently used in animal studies on vascular and neuronal functions [24, 25]. Intra-arterial application of α,β-meATP in cats stimulated preferably C and slowly conducting Aβ fibres of triceps surae muscle [26] and caused inward currents in putative nociceptive dorsal root ganglion neurons [27] and sustained facilitation of the jaw opening reflex in mice as a measure of nociceptive processing in the trigeminal brainstem [28].

The present study was made to explore if electrophysiological recordings from spinal trigeminal neurons may reveal information about the processes underlying trigeminal reflex facilitation and headache associated with activation of pericranial muscle nociceptors. We used an approved model of meningeal nociception to record extracellularly from spinal trigeminal neurons with afferent input from the parietal dura mater, which is regarded to be involved in headache generation. The aim of the study was to find out whether and to which extent α,β-meATP injection into the temporal muscle modulates the neuronal activity of these neurons. The convergence of afferent information to second order neurons in the cervical dorsal horn from the supratentorial dura mater and the greater occipital nerve innervating major parts of neck muscles and skin has earlier been demonstrated [5]. Similar convergent mechanisms should be expected in the trigeminal nucleus caudalis, which is part of the trigeminal brainstem nuclear complex receiving both meningeal and cervical spinal afferent input [29, 30]. However, in the light of recent tracing experiments [31] collateral innervation of meningeal and pericranial structures by same primary trigeminal ganglion neurons may also contribute to the convergence of afferent information from different compartments to spinal trigeminal neurons.

## Methods

The study was performed in accordance with the ethical issues of the International Association for the Study of Pain and with the guidelines and regulations of animal care provided by the Council Directive 2010/63EU of the European Parliament. The protocol was reviewed by an ethics committee and authorized by the local district government.

Sixteen adult male Wistar rats with body weights ranging from 250 to 410 g were used. Rats were bred and held in groups of 2–4 in the Institute’s animal house under a 12/12 h light–dark cycle with food and water ad libitum. They were initially anaesthetised using 4 % isoflurane (Forene, Abbott, Wiesbaden, Germany) administered with an evaporator system (Vapor 19.3, Dräger, Lübeck, Germany) in a closed box. After the initial anaesthesia, 2 % isoflurane administration was maintained through a mask on the animal’s snout. The right femoral artery and vein were catheterized for systemic blood pressure recording and substance administration. Saline supplemented with 1 IU/ml heparin (Heparin-Natrium-5000-ratiopharm, Ulm, Germany) was permanently infused through the arterial catheter (rate 0.2 ml/h) to prevent blood clotting. Subsequently a tracheal tube (intravenous catheter, Vasuflo-T, Dispomed, Gelnhausen, Germany) was inserted in order to ventilate the animals (Rodent Ventilator, Ugo Basile, Comerio VA, Italy) with 2 % isoflurane in oxygen-enriched room air. The expiratory CO_2_ was monitored (Artema MM 200, Karl Heyer, Bad Ems, Germany) and kept steady at 3 % by modulating the ventilation frequency between 70 and 100 strokes per minute to suppress spontaneous breathing. Using a feedback controlled heating pad (TKM 0902, Föhr Medical Instruments, Frankfurt, Germany) and a rectal probe, the animal’s body temperature was maintained at 37–37.5 °C. Mean arterial pressure ranged from 80 to 100 mmHg (Pressure Monitor BP-1, World Precision Instruments, Sarasota, Florida) and was constant within each experiment. Accumulation of viscous mucus in the tracheal tube was prevented by intraperitoneal injection of 0.05 mg/kg atropine sulfate (Braun, Melsungen, Germany). The eyes of the animals were protected by an ointment (Bepanthen, Bayer, Leverkusen, Germany). During recordings the animals were paralyzed with i.v. administration of 50 mg/kg gallamine triethiodide (Sigma, Steinheim, Germany). The narcosis was maintained at constant depth during the experiment so that noxious pinch stimuli did not evoke nociceptive reflexes or changes in arterial blood pressure that may signal pain. After the experiments the animals were euthanized by administering an overdose of thiopental (Trapanal, Nycomed, Konstanz, Germany) intravenously.

### Specific surgery

The animals were placed in a stereotaxic frame and the head fixed using ear bars and a snout clamp. The skull was exposed by cutting the skin along the midline from the forehead to the neck and extending the skin flaps, so that the dorsal part of the temporal muscle was visible. Using a dental drill and saline cooling the right parietal skull was carefully trepanned, thus avoiding dural blood vessel bleeding. After the dura mater was exposed by a cranial window of about 6 × 4 mm, it was protected from drying by cotton pads soaked with 0.9 % saline. In order to gain access to the medullary brainstem, the neck muscles were separated in the midline, detached from their insertions and held apart with a clamp. Finally the atlanto-occipital ligament was cut, exposing the spinal dura underneath.

### Recordings

In order to record neuronal activity from the spinal trigeminal nucleus caudalis, custom-made carbon fibre glass microelectrodes (impendance 0.1–5 MΩ) were inserted into the ipsilateral medulla within the region of the spinal trigeminal nucleus caudalis. A microstepper was used to advance the electrodes through the brainstem at steps of 2.5 μm, while single units with meningeal receptive fields were detected by their firing of action-potentials to mechanical probing of the exposed dura mater with von Frey filaments. Receptive fields in the temporal muscle and neck muscles, facial skin, whiskers, and cornea were located using a fine glass rod. Mechanical stimuli were occasionally applied to the receptive fields to assure that the same neuron was recorded throughout the experiment. The responses to these stimuli were spared in the analysis.

Recorded signals were band-pass filtered (0.5–1 kHz), amplified and processed (CED 1401, Cambridge Electronic Design, Cambridge, UK). Spike-analysis was performed offline using the discharges generated by mechanical stimulation of meningeal receptive fields as templates (Spike 2 software application, Cambridge Electronic Design). At the end of the experiment the x-y-position of the recording site relative to the caudal extension of the obex was noted according to the readings of the microdrives.

### Experimental protocols

When the ongoing (unstimulated) neuronal activity of a unit was visibly stable, it was recorded for a control period of 30 min without any treatment (baseline activity). Subsequently, saline (0.9 % NaCl, 50 μl) was injected with a U-100 insulin syringe (BD, Franklin Lakes, NJ, USA) deep into the temporal muscle close to the crista temporalis within one minute. After another 30 min of recording, 50 μl of 10 mM α,β-meATP (α,β-methylene adenosine 5′-triphosphate disodium salt hydrate, Sigma-Aldrich, Taufkirchen, Germany) was injected into the temporal muscle and the recording continued for 60 min. After another 10 min without further treatment (new baseline), in most of the experiments 60 μl of 2 % lidocaine (Xylocain, Astra Zeneca, Wedel, Germany) was injected into the ipsilateral medial (semispinalis and rectus capitis) neck muscles followed by a recording period of 20 min. Next the temporal muscle was injected with 60 μl lidocaine and the activity recorded for another 20 min. Finally, 60 μl of lidocaine was applied onto the exposed cranial dura mater followed by a final recording period of 20 min. In some experiments 60 μl of lidocaine was injected only into the ipsilateral temporal muscle immediately followed by 50 μl of α,β-meATP, then the recording was continued for 60 min.

### Data analysis

Discharges per minute were counted throughout the experiments. For comparison of data the neuronal activity was averaged across 10 min intervals, the injection minutes were excluded from analysis. Statistical analysis was made with Statistica software 7.1 (Statsoft GmbH, Hamburg, Germany). Data were analysed using analysis of variance (ANOVA) with repeated measurements followed by Fisher’s least significant difference (LSD) post hoc test comparing the averaged activity within intervals of 10 and 30 min. Differences were considered significant at *p* ≤ 0.05.

## Results

### General properties of neurons

The recording sites of 16 units were located 1.64–3.4 mm caudal to the obex and 0.35–1.64 mm lateral to the midline and at depths of 302–972 μm below the dorsal surface of the medulla. The ongoing activity of units varied between 0.1 and 1304 (mean 183, SEM ± 83) spikes per minute during the control period. The meningeal receptive fields, mostly located close to the middle meningeal artery, were mapped. The mechanical threshold determined with graded von Frey filaments ranged from 0.49 to 11.8 mN (mean 5.0 mN). All recorded units received also convergent input from facial areas in the ophthalmic, maxillary or mandibular division of the trigeminal nerve as well as from the temporal muscle and/or the neck muscles and the periosteum around the cranial window and were characterized as wide-dynamic range (WDR) units according to their responses to touch stimuli of the facial skin. Mechanical threshold of muscle input ranged from 8.8 to 14.7 mN (mean temporal muscle 12.7 mN, neck muscles 13.6 mN).

The units were activated by electrical pulses (duration 1 ms) with thresholds ranging from 0.4 to 2.1 mA applied to the meningeal receptive field. The latencies after a single electrical pulse close above threshold ranged from 12 to 25 ms. Taking a distance of 25 mm from the dura mater to the caudal medulla the units received afferent input from meningeal slowly conducting Aδ and/or C-fibres.

### Effects of vehicle and α,β-meATP

**Fig. 1:**
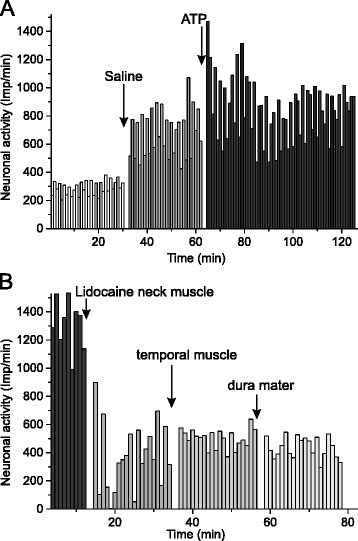
Typical experiment showing the ongoing activity of a spinal trigeminal neuron displayed in discharges per minute. Injection of saline and α, β-meATP into the ipsilateral temporal muscle increased the activity (**a**), which was reversed by injection of lidocaine into the neck muscles (**b**), while lidocaine injection into the temporal muscle had no additional effect in this case

**Fig. 2:**
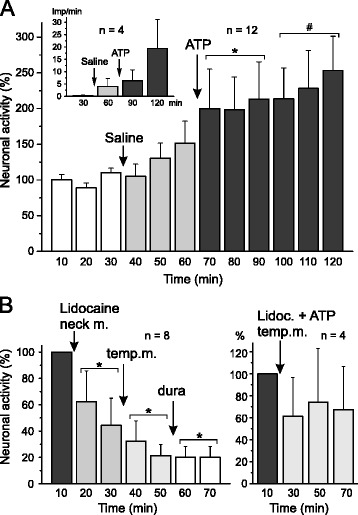
**a** Activity of spinal trigeminal neurons with afferent input from the dura mater and the temporal muscle. The normalized activity displayed in 10 min intervals increased more and more after injection of vehicle (saline) and α,β-meATP (ATP) into the ipsilateral temporal muscle (* significant difference to baseline, # to baseline and intervals after vehicle). The inset shows four additional units (activity displayed in 30 min intervals) which did not fit to the normalized sample because of their low spontaneous activity and relatively high activation following α,β-meATP injection. **b** Activity of spinal trigeminal neurons pre-treated by injection of α,β-meATP into the ipsilateral temporal muscle (experiments continued from (**a**)). Left: The activity (normalized to the 10 min interval following the experiment in (**a**)) is significantly (*) reduced after injection of lidocaine into the occipital muscles and further after injection into the temporal muscle (left) but not more after application of lidocaine onto the dura mater. Right: Four additional units recorded during lidocaine injection only into the temporal muscle, three of them showing decreased activity (displayed in 20 min intervals)

After a control period of 30 min (baseline) vehicle was slowly injected into the temporal muscle, which was not followed by a significant increase in activity in the whole sample of 16 units within the recording time of 30 min, though the activity increased visibly in five (see example Fig. 1a) and decreased in 3 units. After the injection of α,β-meATP, the activity in half of the sample increased within 10 min and stayed at a higher level (Fig. 1a), in the other half it increased more or less gradually within the following 30 min. Four units of the sample showed virtually no activity (< 2 spikes/min) at baseline; two of them were only transiently activated during injection of saline and/or α,β-meATP ending up with no activity at 50 min after the injection. Repeated measures ANOVA applied to all 16 units during the 30 min periods before and after the injection of substances indicated a significant difference (*F*_3,45_ = 3.8; *p* < 0.02). Posthoc analysis revealed that the activity within the first 30 min after α,β-meATP was significantly different to the baseline (*p* = 0.01), while the activity within the period of 30–60 min after α,β-meATP was different to the baseline (*p* < 0.01) and to the activity after the vehicle injection (*p* < 0.05). Analysis of 10 min intervals of the sample of 12 units with spontaneous activity revealed the same differences (Fig. 2a).

### Effects of lidocaine

In 12 units the experiment was continued with local anaesthesia. After 60 min of recording following α,β-meATP injection, the mean activity within 10 min (new baseline) ranged from 20 to 1357 (mean 425, SEM ± 129) spikes/min. To test if the spinal trigeminal activity induced by local α,β-meATP could be blocked and reversed by anaesthesia of the homologous muscle, lidocaine was injected into the temporal muscle immediately followed by injection of α,β-meATP in four of the experiments (Fig. 2b right). In two units the neuronal activity decreased dramatically after the injections and did not recover during the recording time of further 60 min. In one unit the activity was slightly decreased, in another unit increased.

In the remaining 8 experiments the effect of local anaesthesia of the heterologous structures delivering convergent input to the activated spinal trigeminal neurons was tested. Lidocaine was injected first into the neck muscles, 20 min later into the temporal muscle and after another 20 min finally applied onto the dura mater (Figs. 1b, 2b left). In all units but two the activity decreased already after the first lidocaine injection and continued to decrease in the whole sample of units (repeated measures ANOVA, *F*_3,21_ = 5.1; *p* < 0.01). Posthoc analysis revealed significant differences to the baseline after each lidocaine application (*p* < 0.01) (Fig. 2b left).

## Discussion

The present experiments show that spinal trigeminal neurons with afferent input from the temporal muscle and the cranial dura mater increase their ongoing activity after injecting α,β-meATP deeply into the temporal muscle. This activation developed gradually in half of the neurons. The injection itself may have contributed to the increased activity in some neurons, likely caused by mechanical irritation, but did not play a significant role in the whole sample of units. The activating effect of α,β-meATP was persistent and even increased further beyond the 60 min after injection into the temporal muscle, before local anaesthesia was applied. Lidocaine injection into this muscle had a dramatical effect in lowering the activity of spinal trigeminal neurons suggesting that this activity was mainly maintained by afferent input from the temporal muscle. However, this could have been expected, while the main question was whether or not the ongoing activity was also dependent on the afferent input from the other structures, in which receptive fields were located. Therefore we anaesthetized first the neck muscles and later the dura mater in the main sample of units. A rapid decrease in α,β-meATP-driven activity was seen after anaesthesia of the neck muscles, indicating significant convergent input in accordance with the location of receptive fields. This clear effect could be explained by assuming that the afferents supplying the neck muscles had been sensitized during surgery (exposure and separation of muscles, dissection of the atlanto-occipital ligament) causing ongoing afferent activity that contributed to maintain the activity of second order neurons. After additional anaesthesia of the temporal muscle the activity further declined but local anaesthesia of the dura mater was not further effective, because the activity had already returned to the basal level. It seems that removal of a critical portion of afferent input to these multireceptive units is sufficient to restore their basal activity. This is reminiscent of previous recordings in which the ongoing central activity was blocked by anaesthesia of the trigeminal ganglion [32].

The activity evoked by α,β-meATP was probably due to purinergic signaling involving P2X receptor channels. Functional experiments indicate the preference of P2X receptors in skeletal muscle nociception. Activation of P2X3 receptors caused hyperalgesia of rat masseter muscle, which involved phosphorylation of TRPV1 receptors [33]. A variety of experiments investigating craniofacial nociceptive reflexes revealed various purinergic mechanisms in neck muscle nociception including P2X receptor activation that drive the nociceptive input to the trigeminal brainstem [6, 34]. The relevance of this translational model of tension-type headache is underlined by the suppression of α,β-meATP-induced facilitation of neck muscle nociception through inhibition of nitric oxide generation and acetylsalicylic acid [35, 36].

Activation of ATP-sensitive P2X receptors evoked also skeletal muscle afferent-mediated pressor responses [37]. Moreover, activation of P2X receptors in orofacial tissues have been found to play a critical role in central sensitization of second order neurons in the spinal trigeminal nucleus [38]. The fact that the elevated spinal trigeminal activity in our experiments could be normalized with the abrogation of peripheral input may suggest that central sensitization was not yet fully developed.

Spinal trigeminal neurons with meningeal afferent input have frequently been recorded to study mechanisms of meningeal nociception [39, 40], though many of these neurons receive convergent cutaneous afferent input and show a wide-dynamic range (WDR) character, i.e. respond to cutaneous stimuli of increasing strength in a graded fashion [41, 42]. The units recorded in this study can be regarded to signal muscle pain, since they are activated by pressure onto the temporal muscle and respond to the noxious chemical stimulus α,β-meATP as a surrogate of high extracellular concentrations of ATP. In addition, due to their meningeal receptive input, they may signal meningeal nociceptive events, and respective neurons in humans may be those signaling head pain. Earlier, Bartsch and Goadsby [5] recorded from second-order neurons in the rat dorsal horn of the C2 cervical segment with afferent input from the supratentorial dura mater and the greater occipital nerve, which innervates major parts of the neck muscles and skin. Electrical stimulation of the greater occipital nerve as well as application of the TRPA1/V1 agonist mustard oil to the dura or the muscle enhanced the response of most of the spinal neurons to electrical stimuli applied to the dura or the nerve, revealing convergent input from both structures to the spinal trigeminal second order neurons. Neurons were also sensitized by this treatment showing decreased mechanical thresholds of the dural receptive sites and enlarged facial and cervical cutaneous mechanoreceptive fields as well as increased responses to noxious mechanical stimulation of paraspinal muscles [43]. These results, as well as the data of our study, may reveal a pathophysiological basis for pain referred from meningeal to cervical innervations territories, which may also underlie the clinical phenomena of cervical hypersensitivity and neck muscle tension in primary headaches like migraine [44]. Conversely, the convergent nociceptive input from neck muscles and meninges may form the basis for tension-type headache which can be induced or aggravated by muscle tension. Extracellular accumulation of ATP, which is measurably increased during experimental muscle tension and compression [15, 16] and intense natural muscle contraction [17], may contribute to this pathology.

Recent findings extended the concept of afferent convergence to second order neurons in the trigeminal brainstem complex. Through histological examinations, tracing studies and electrophysiological recordings in rodent and human calvaria convincing evidence was collected showing that the periosteum and deep layers of pericranial muscles including the temporal muscle are innervated by collaterals of meningeal afferents that traverse the dura mater and project through fissures and emissary canals of the skull to these extracranial tissues [31, 45]. Several of these single afferents could be activated both from the inside and the outside of the rat skull, noxious stimulation of the temporal muscle caused neuropeptide release from the dura mater, and collision experiments proved that at least some of these intra- and extracranial afferent fibres belong to same afferent neurons [46]. Thus, in addition to convergent afferent input, the second order neurons processing muscle pain may be driven by primary nociceptive afferents that innervate both the dura mater and the temporal muscle. Recently there is evidence that also neck muscles can be supplied by collaterals of meningeal afferents, since retrograde tracing from neck muscles and the dura mater of the occipital cranial fossa showed convergence of the tracer in some trigeminal ganglion neurons (unpublished data from our group).

Inhibition of the afferent muscle input could be rather effective in reducing the activity of the central neurons, as evidenced in the present study using local injection of anesthetics into the temporal muscle. Similarly, afferent nerve blockade such as anaesthesia of the greater occipital nerve has been proposed to reduce nociceptive input and hence headache and has been used with equivocal success in primary headaches [47, 48] but is recently used as a therapeutic option in refractory headache [49], cluster headache [50] and chronic migraine [51]. This concept is supported by clinical experiences and therapeutic efforts in tension-type headache and other primary headaches, which aim at decreasing muscle tension and nociceptive input from pericranial muscles [52, 53].

## Conclusions

Injection of α,β-meATP into the temporal muscle in rat induces ongoing activity of spinal trigeminal neurons with meningeal receptive fields and local anaesthesia of single neck muscles and/or the temporal muscle can abolish this activation. This shows that distinct spinal trigeminal neurons processing meningeal nociceptive information are under the control of convergent afferent input from several pericranial muscles, which may already occur at the primary afferent level by collaterals of meningeal nociceptors innervating pericranial muscle compartments. Blockade of one of these inputs can normalize central trigeminal activity providing an explanation for the beneficial effect of nerve blockade and extracranial therapeutic manipulations in primary headaches.

## Abbreviations

ANOVA: Analysis of variance
ATP: Adenosine 5′-triphosphate
LSD test: Fisher’s least significant difference test
TTH: Tension-type headache
α,β-meATP: α,β-methylene adenosine 5′-triphosphate
